# A new *S*-type eigenvalue inclusion set for tensors and its applications

**DOI:** 10.1186/s13660-016-1200-3

**Published:** 2016-10-19

**Authors:** Zheng-Ge Huang, Li-Gong Wang, Zhong Xu, Jing-Jing Cui

**Affiliations:** Department of Applied Mathematics, Northwestern Polytechnical University, Xi’an, Shaanxi 710072 P.R. China

**Keywords:** 15A18, 15A69, tensor eigenvalue, nonsingular *M*-tensor, minimum *H*-eigenvalue, nonnegative tensor, spectral radius, positive definite

## Abstract

In this paper, a new *S*-type eigenvalue localization set for a tensor is derived by dividing $N=\{1,2,\ldots,n\}$ into disjoint subsets *S* and its complement. It is proved that this new set is sharper than those presented by Qi (J. Symb. Comput. 40:1302-1324, 2005), Li *et al.* (Numer. Linear Algebra Appl. 21:39-50, 2014) and Li *et al.* (Linear Algebra Appl. 481:36-53, 2015). As applications of the results, new bounds for the spectral radius of nonnegative tensors and the minimum *H*-eigenvalue of strong *M*-tensors are established, and we prove that these bounds are tighter than those obtained by Li *et al.* (Numer. Linear Algebra Appl. 21:39-50, 2014) and He and Huang (J. Inequal. Appl. 2014:114, 2014).

## Introduction

Eigenvalue problems of higher order tensors have become an important topic in the applied mathematics branch of numerical multilinear algebra, and they have a wide range of practical applications, such as best-rank one approximation in data analysis [5], higher order Markov chains [6], molecular conformation [7], and so forth. In recent years, tensor eigenvalues have caused concern of lots of researchers [1, 3, 4, 8–20].

One of many practical applications of eigenvalues of tensors is that one can identify the positive (semi-)definiteness for an even-order real symmetric tensor by using the smallest *H*-eigenvalue of a tensor, consequently, one can identify the positive (semi-)definiteness of the multivariate homogeneous polynomial determined by this tensor; for details, see [1, 21, 22].

However, as mentioned in [21, 23, 24], it is not easy to compute the smallest *H*-eigenvalue of tensors when the order and dimension are very large, we always try to give a set including all eigenvalues in the complex. Some sets including all eigenvalues of tensors have been presented by some researchers [1–3, 21–24]. In particular, if one of these sets for an even-order real symmetric tensor is in the right-half complex plane, then we can conclude that the smallest *H*-eigenvalue is positive, consequently, the corresponding tensor is positive definite. Therefore, the main aim of this paper is to study the new eigenvalue inclusion set for tensors called the new *S*-type eigenvalue inclusion set, which is sharper than some existing ones.

For a positive integer *n*, *N* denotes the set $N=\{1,2,\ldots,n\}$. The set of all real numbers is denoted by $\mathbb{R}$, and $\mathbb{C}$ denotes the set of all complex numbers. Here, we call $\mathcal {A}=(a_{i_{1}\cdots i_{m}})$ a complex (real) tensor of order *m* dimension *n*, denoted by $\mathbb{C}^{[m,n]}(\mathbb{R}^{[m,n]})$, if $a_{i_{1}\cdots i_{m}}\in{\mathbb{C}}(\mathbb{R})$, where $i_{j}\in{N}$ for $j=1,2,\ldots,m$ [23].

Let $\mathcal{A}\in\mathbb{R}^{[m,n]}$, and $x\in{\mathbb{C}}^{n}$. Then

$$ \mathcal{A}x^{m-1}:= \Biggl(\sum_{i_{2},\ldots, i_{m}=1}^{n}a_{ii_{2}\cdots i_{m}}x_{i_{2}} \cdots x_{i_{m}} \Biggr)_{1\leq {i}\leq{n}}, $$

a pair $(\lambda,x)\in{\mathbb{C}}\times(\mathbb{C}^{n}/\{0\})$ is called an eigenpair of $\mathcal{A}$ [18] if

$$ \mathcal{A}x^{m-1}=\lambda x^{[m-1]}, $$

where $x^{[m-1]}=(x_{1}^{m-1},x_{2}^{m-1},\ldots,x_{n}^{m-1})^{T}$ [25]. Furthermore, we call $(\lambda,x)$ an *H*-eigenpair, if both *λ* and *x* are real [1].

A real tensor of order *m* dimension *n* is called the unit tensor [21], denoted by $\mathcal{I}$, if its entries are $\delta_{i_{1}\cdots i_{m}}$ for $i_{1},\ldots, i_{m}\in{N}$, where

$$ \delta_{i_{1}\cdots i_{m}}=\left \{ \textstyle\begin{array}{l@{\quad}l} 1, &\text{if } i_{1}=\cdots=i_{m}, \\ 0, & \text{otherwise}. \end{array}\displaystyle \right . $$

An *m*-order *n*-dimensional tensor $\mathcal{A}$ is called nonnegative [9, 10, 13, 14, 26], if each entry is nonnegative. We call a tensor $\mathcal{A}$ a *Z*-tensor, if all of its off-diagonal entries are non-positive, which is equivalent to writing $\mathcal{A}=s\mathcal {I}-\mathcal{B}$, where $s>0$ and $\mathcal{B}$ is a nonnegative tensor ($\mathcal{B}\geq{0}$), denoted by $\mathbb{Z}$ the set of *m*-order and *n*-dimensional *Z*-tensors. A *Z*-tensor $\mathcal{A}=s\mathcal{I}-\mathcal{B}$ is an *M*-tensor if $s\geq\rho (\mathcal{B})$, and it is a nonsingular (strong) *M*-tensor if $s>\rho (\mathcal{B})$ [20, 27].

The tensor $\mathcal{A}$ is called reducible if there exists a nonempty proper index subset $\mathbb{J}\subset N$ such that $a_{i_{1}i_{2}\cdots i_{m}}=0$, $\forall i_{1}\in\mathbb{J}$, $\forall i_{2},\ldots,i_{m}\notin\mathbb{J}$. If $\mathcal{A}$ is not reducible, then we call $\mathcal{A}$ is irreducible [19]. The spectral radius $\rho(\mathcal{A})$ [14] of the tensor $\mathcal {A}$ is defined as

$$ \rho(\mathcal{A})=\max\bigl\{ \vert \lambda \vert :\lambda\text{ is an eigenvalue of } \mathcal{A}\bigr\} . $$

Denote by $\tau(\mathcal{A})$ the minimum value of the real part of all eigenvalues of the nonsingular *M*-tensor $\mathcal{A}$ [4]. A real tensor $\mathcal{A}=(a_{i_{1}\cdots i_{m}})$ is called symmetric [1–3, 13, 22, 23] if

$$ a_{i_{1}\cdots i_{m}}=a_{\pi(i_{1}\cdots i_{m})},\quad \forall\pi\in\Pi _{m}, $$

where $\Pi_{m}$ is the permutation group of *m* indices.

Let $\mathcal{A}=(a_{i_{1}\cdots i_{m}})\in{\mathbb{R}}^{[m,n]}$. For $i,j\in{N}$, $j\neq{i}$, denote

$$\begin{aligned}& R_{i}(\mathcal{A})=\sum_{i_{2},\ldots, i_{m}=1}^{n}a_{ii_{2}\cdots i_{m}}, \qquad R_{\mathrm{max}}(\mathcal{A})=\max_{i\in{N}}R_{i}( \mathcal{A}),\qquad R_{\mathrm{min}}(\mathcal{A})=\min_{i\in{N}}R_{i}( \mathcal{A}), \\& r_{i}(\mathcal{A})=\sum_{\delta_{ii_{2}\cdots i_{m}}=0}|a_{ii_{2}\cdots i_{m}}|, \qquad r_{i}^{j}(\mathcal{A})=\sum _{\substack{\delta_{ii_{2}\cdots i_{m}}=0,\\ \delta_{ji_{2}\cdots i_{m}}=0}}|a_{ii_{2}\cdots i_{m}}|=r_{i}(\mathcal{A})-|a_{ij\cdots {j}}|. \end{aligned}$$

Recently, much literature has focused on the bounds of the spectral radius of nonnegative tensor in [2, 3, 14, 15, 17–19, 24, 28]. In addition, in [4], He and Huang obtained the upper and lower bounds for the minimum *H*-eigenvalue of nonsingular *M*-tensors. Wang and Wei [16] presented some new bounds for the minimum *H*-eigenvalue of nonsingular *M*-tensors, and they showed those are better than the ones in [4] in some cases. As applications of the new *S*-type eigenvalue inclusion set, the other main results of this paper is to provide sharper bounds for the spectral radius of nonnegative tensors and the minimum *H*-eigenvalue of nonsingular *M*-tensors, which improve some existing ones.

Before presenting our results, we review the existing results that relate to the eigenvalue inclusion sets for tensors. In 2005, Qi [1] generalized the Geršgorin eigenvalue inclusion theorem from matrices to real supersymmetric tensors, which can be easily extended to general tensors [2, 13].

### Lemma 1.1

[1]

*Let* $\mathcal{A}=(a_{i_{1}\cdots i_{m}})\in{\mathbb{C}}^{[m,n]}$, $n\geq {2}$. *Then*

$$ \sigma(\mathcal{A})\subseteq\Gamma(\mathcal{A})=\bigcup _{i\in{N}}\Gamma _{i}(\mathcal{A}), $$

*where* $\sigma(\mathcal{A})$ *is the set of all the eigenvalues of* $\mathcal{A}$ *and*

$$ \Gamma_{i}(\mathcal{A})=\bigl\{ z\in{\mathbb{C}}:|z-a_{i\cdots i}| \leq r_{i}(\mathcal{A})\bigr\} . $$

To get sharper eigenvalue inclusion sets than $\Gamma(\mathcal{A})$, Li *et al.* [2] extended the Brauer eigenvalue localization set of matrices [29, 30] and proposed the following Brauer-type eigenvalue localization sets for tensors.

### Lemma 1.2

[2]

*Let* $\mathcal{A}=(a_{i_{1}\cdots i_{m}})\in{\mathbb{C}}^{[m,n]}$, $n\geq {2}$. *Then*

$$ \sigma(\mathcal{A})\subseteq\mathcal{K}(\mathcal{A})=\bigcup _{i,j\in {N},j\neq{i}}\mathcal{K}_{i,j}(\mathcal{A}), $$

*where*

$$ \mathcal{K}_{i,j}(\mathcal{A})=\bigl\{ z\in{\mathbb{C}}:\bigl(\vert z-a_{i\cdots i}\vert -r_{i}^{j}(\mathcal{A}) \bigr)|z-a_{j\cdots j}|\leq|a_{ij\cdots j}|r_{j}(\mathcal{A})\bigr\} . $$

In addition, in order to reduce computations of determining the sets $\sigma(\mathcal{A})$, Li *et al.* [2] also presented the following *S*-type eigenvalue localization set by breaking *N* into disjoint subsets *S* and *S̄*, where *S̄* is the complement of *S* in *N*.

### Lemma 1.3

[2]

*Let* $\mathcal{A}=(a_{i_{1}\cdots i_{m}})\in{\mathbb{C}}^{[m,n]}$, $n\geq {2}$, *and* *S* *be a nonempty proper subset of N*. *Then*

$$ \sigma(\mathcal{A})\subseteq\mathcal{K}^{S}(\mathcal{A})= \biggl( \bigcup_{i\in{S},j\in{\bar{S}}}\mathcal{K}_{i,j}(\mathcal{A}) \biggr)\cup \biggl(\bigcup_{i\in{\bar{S}},j\in{{S}}}\mathcal{K}_{i,j}( \mathcal {A}) \biggr), $$

*where* $\mathcal{K}_{i,j}(\mathcal{A})$ ($i\in{S}$, $j\in{\bar{S}}$ *or* $i\in{\bar{S}}$, $j\in{{S}}$) *is defined as in Lemma* 1.2.

Based on the results of [2], in the sequel, Li *et al.* [3] exhibited a new tensor eigenvalue inclusion set, which is proved to be tighter than the sets in Lemma 1.2.

### Lemma 1.4

[3]

*Let* $\mathcal{A}=(a_{i_{1}\cdots i_{m}})\in{\mathbb{C}}^{[m,n]}$, $n\geq {2}$, *and* *S* *be a nonempty proper subset of N*. *Then*

$$ \sigma(\mathcal{A})\subseteq\Delta(\mathcal{A})=\bigcup _{i,j\in {N},j\neq{i}}\Delta_{i}^{j}(\mathcal{A}), $$

*where*

$$ \Delta_{i}^{j}(\mathcal{A}) = \bigl\{ z\in{\mathbb{C}}:\bigl\vert (z-a_{i\cdots i}) (z-a_{j\cdots j})-a_{ij\cdots j}a_{ji\cdots i} \bigr\vert \leq|z-a_{j\cdots j}|r_{i}^{j}(\mathcal {A})+|a_{ij\cdots j}|r_{j}^{i}(\mathcal{A}) \bigr\} . $$

In this paper, we continue this research on the eigenvalue inclusion sets for tensors; inspired by the ideas of [2, 3], we obtain a new *S*-type eigenvalue inclusion set for tensors. It is proved to be tighter than the tensor Geršgorin eigenvalue inclusion set $\Gamma(\mathcal{A})$ in Lemma 1.1, the Brauer eigenvalue localization set $\mathcal{K}(\mathcal{A})$ in Lemma 1.2, the *S*-type eigenvalue localization set $\mathcal{K}^{S}(\mathcal{A})$ in Lemma 1.3, and the set $\Delta(\mathcal{A})$ in Lemma 1.4. As applications, we establish some new bounds for spectral radius of nonnegative tensors and the minimum *H*-eigenvalue of strong *M*-tensors. Numerical examples are implemented to illustrate this fact.

The remainder of this paper is organized as follows. In Section 2, we recollect some useful lemmas on tensors which are utilized in the next sections. In Section 3.1, a new *S*-type eigenvalue inclusion set for tensors is given, and proved to be tighter than the existing ones derived in Lemmas 1.1-1.4. Based on the results of Section 3.1, we propose a new upper bound for the spectral radius of nonnegative tensors in Section 3.2; comparison results for this new bound and that derived in [2] are also investigated in this section. Section 3.3 is devoted to the exhibition of new upper and lower bounds for the minimum *H*-eigenvalue of strong *M*-tensors, which are proved to be sharper than the ones obtained by He and Huang [4]. Finally, some concluding remarks are given to end this paper in Section 4.

## Preliminaries

In this section, we start with some lemmas on tensors. They will be useful in the following proofs.

### Lemma 2.1

[16]

*If* $\mathcal{A}\in{\mathbb{R}}^{[m,n]}$ *is irreducible nonnegative*, *then* $\rho(\mathcal{A})$ *is a positive eigenvalue with an entrywise positive eigenvector* *x*, *i*.*e*., $x>0$, *corresponding to it*.

### Lemma 2.2

[2]

*Let* $\mathcal{A}\in{\mathbb{R}}^{[m,n]}$ *be a nonnegative tensor*. *Then* $\rho(\mathcal{A})\geq\max_{i\in{N}}\{a_{i\cdots i}\}$.

### Lemma 2.3

[13]

*Suppose that* $0\leq\mathcal{A}<\mathcal{C}$. *Then* $\rho(\mathcal{A})\leq \rho(\mathcal{C})$.

### Lemma 2.4

[4]

*Let* $\mathcal{A}$ *be a strong* *M*-*tensor and denoted by* $\tau(\mathcal {A})$ *the minimum value of the real part of all eigenvalues of* $\mathcal {A}$. *Then* $\tau(\mathcal{A})$ *is an eigenvalue of* $\mathcal{A}$ *with a nonnegative eigenvector*. *Moreover*, *if* $\mathcal{A}$ *is irreducible*, *then* $\tau(\mathcal{A})$ *is a unique eigenvalue with a positive eigenvector*.

### Lemma 2.5

[4]

*Let* $\mathcal{A}$ *be an irreducible strong* *M*-*tensor*. *Then* $\tau(\mathcal {A})\leq\min_{i\in{N}}\{a_{i\cdots i}\}$.

### Lemma 2.6

[20]

*A tensor* $\mathcal{A}$ *is semi*-*positive if and only if there exists* $x\geq{0}$ *such that* $\mathcal{A}x^{m-1}>0$.

### Lemma 2.7

[20]

*A* *Z*-*tensor is a nonsingular* *M*-*tensor if and only if it is semi*-*positive*.

### Lemma 2.8

[4]

*Let* $\mathcal{A},\mathcal{B}\in{\mathbb{Z}}$, *assume that* $\mathcal{A}$ *is an* *M*-*tensor and* $\mathcal{B}\geq{\mathcal{A}}$. *Then* $\mathcal{B}$ *is an* *M*-*tensor*, *and* $\tau(\mathcal{A})\leq\tau(\mathcal{B})$.

## Main results

### A new *S*-type eigenvalue inclusion set for tensors

In this section, we propose a new *S*-type eigenvalue set for tensors and establish the comparisons between this new set with those in Lemmas 1.1-1.4.

#### Theorem 3.1

*Let* $\mathcal{A}=(a_{i_{1}\cdots i_{m}})\in{\mathbb{C}}^{[m,n]}$ *with* $n\geq{2}$. *And let* *S* *be a nonempty proper subset of* *N*. *Then*

1 $$ \sigma(\mathcal{A})\subseteq\Upsilon^{S}(\mathcal{A}):= \biggl(\bigcup_{i\in{S},j\in{\bar{S}}}\Upsilon_{i}^{j}( \mathcal{A}) \biggr)\cup \biggl(\bigcup_{i\in{\bar{S}},j\in{S}} \Upsilon_{i}^{j}(\mathcal {A}) \biggr), $$

*where*

$$ \Upsilon_{i}^{j}(\mathcal{A}) = \bigl\{ z\in{\mathbb{C}}: \bigl\vert (z-a_{i\cdots i}) (z-a_{j\cdots j})-a_{ij\cdots j}a_{ji\cdots i} \bigr\vert \leq \vert z-a_{j\cdots j}\vert r_{i}^{j}( \mathcal {A})+\vert a_{ij\cdots j}\vert r_{j}^{i}( \mathcal{A}) \bigr\} . $$

#### Proof

For any $\lambda\in\sigma(\mathcal{A})$, let $x=(x_{1},\ldots,x_{n})^{T}\in{\mathbb{C}}^{n}/\{0\}$ be an eigenvector corresponding to *λ*, *i.e.*,

2 $$ \mathcal{A}x^{m-1}=\lambda x^{[m-1]}. $$

Let $|x_{p}|=\max_{i\in{S}}\{|x_{i}|\}$ and $|x_{q}|=\max_{i\in{\bar {S}}}\{|x_{i}|\}$. Then, $x_{p}\neq{0}$ or $x_{q}\neq{0}$. Now, let us distinguish two cases to prove.

(i) $|x_{p}|\geq|x_{q}|$, so $|x_{p}|=\max_{i\in{N}}\{|x_{i}|\}$ and $|x_{p}|>0$. For any $j\in{\bar{S}}$, it follows from (2) that

$$ \left \{ \textstyle\begin{array}{l} \sum_{i_{2},\ldots, i_{m}=1}^{n}a_{pi_{2}\cdots i_{m}}x_{i_{2}}\cdots x_{i_{m}}=\lambda x_{p}^{m-1}, \\ \sum_{i_{2},\ldots, i_{m}=1}^{n}a_{ji_{2}\cdots i_{m}}x_{i_{2}}\cdots x_{i_{m}}=\lambda x_{j}^{m-1}. \end{array}\displaystyle \right . $$

Hence, we have

$$ \left \{ \textstyle\begin{array}{l} \sum_{\substack{\delta_{pi_{2}\cdots i_{m}=0}, \\ \delta _{ji_{2}\cdots i_{m}=0}}}a_{pi_{2}\cdots i_{m}}x_{i_{2}}\cdots x_{i_{m}}+a_{p\cdots{p}}x_{p}^{m-1}+a_{pj\cdots{j}}x_{j}^{m-1}=\lambda x_{p}^{m-1}, \\ \sum_{\substack{\delta_{ji_{2}\cdots i_{m}=0}, \\ \delta _{pi_{2}\cdots i_{m}=0}}}a_{ji_{2}\cdots i_{m}}x_{i_{2}}\cdots x_{i_{m}}+a_{j\cdots{j}}x_{j}^{m-1}+a_{jp\cdots{p}}x_{p}^{m-1}=\lambda x_{j}^{m-1}, \end{array}\displaystyle \right . $$

*i.e.*,

3 $$ \left \{ \textstyle\begin{array}{l} \sum_{\substack{\delta_{pi_{2}\cdots i_{m}=0}, \\ \delta _{ji_{2}\cdots i_{m}=0}}}a_{pi_{2}\cdots i_{m}}x_{i_{2}}\cdots x_{i_{m}}=(\lambda-a_{p\cdots{p}})x_{p}^{m-1}-a_{pj\cdots {j}}x_{j}^{m-1}, \\ \sum_{\substack{\delta_{ji_{2}\cdots i_{m}=0}, \\ \delta _{pi_{2}\cdots i_{m}=0}}}a_{ji_{2}\cdots i_{m}}x_{i_{2}}\cdots x_{i_{m}}=(\lambda-a_{j\cdots{j}})x_{j}^{m-1}-a_{jp\cdots {p}}x_{p}^{m-1}. \end{array}\displaystyle \right . $$

Premultiplying by $(\lambda-a_{j\cdots{j}})$ in the first equation of (3) results in

4 $$\begin{aligned}& (\lambda-a_{j\cdots{j}})\sum_{\substack{\delta_{pi_{2}\cdots i_{m}=0}, \\ \delta_{ji_{2}\cdots i_{m}=0}}}a_{pi_{2}\cdots i_{m}}x_{i_{2}} \cdots x_{i_{m}} \\& \quad = (\lambda-a_{j\cdots{j}}) (\lambda-a_{p\cdots {p}})x_{p}^{m-1}-a_{pj\cdots{j}}( \lambda-a_{j\cdots{j}})x_{j}^{m-1}. \end{aligned}$$

Combining (4) and the second equation of (3) one derives

$$\begin{aligned}& (\lambda-a_{j\cdots{j}})\sum_{\substack{\delta_{pi_{2}\cdots i_{m}=0}, \\ \delta_{ji_{2}\cdots i_{m}=0}}}a_{pi_{2}\cdots i_{m}}x_{i_{2}} \cdots x_{i_{m}} +a_{pj\cdots j}\sum_{\substack{\delta_{ji_{2}\cdots i_{m}=0}, \\ \delta _{pi_{2}\cdots i_{m}=0}}}a_{ji_{2}\cdots i_{m}}x_{i_{2}} \cdots x_{i_{m}} \\& \quad = (\lambda-a_{j\cdots{j}}) (\lambda-a_{p\cdots {p}})x_{p}^{m-1}-a_{pj\cdots{j}}a_{jp\cdots{p}}x_{p}^{m-1} \\& \quad = \bigl[(\lambda-a_{j\cdots{j}}) (\lambda-a_{p\cdots{p}})-a_{pj\cdots {j}}a_{jp\cdots{p}} \bigr]x_{p}^{m-1}. \end{aligned}$$

Taking absolute values and using the triangle inequality yield

$$\begin{aligned}& \bigl\vert (\lambda-a_{j\cdots{j}}) (\lambda-a_{p\cdots{p}})-a_{pj\cdots {j}}a_{jp\cdots{p}} \bigr\vert \vert x_{p}\vert ^{m-1} \\& \quad \leq \vert \lambda-a_{j\cdots j}\vert r_{p}^{j}( \mathcal {A})\vert x_{p}\vert ^{m-1}+\vert a_{pj\cdots j}\vert r_{j}^{p}(\mathcal {A})\vert x_{p}\vert ^{m-1}. \end{aligned}$$

Note that $|x_{p}|>0$, thus

5 $$ \bigl\vert (\lambda-a_{j\cdots{j}}) (\lambda-a_{p\cdots{p}})-a_{pj\cdots {j}}a_{jp\cdots{p}} \bigr\vert \leq \vert \lambda-a_{j\cdots j}\vert r_{p}^{j}( \mathcal{A})+\vert a_{pj\cdots j}\vert r_{j}^{p}( \mathcal{A}), $$

which implies that $\lambda\in\Upsilon_{p}^{j}(\mathcal{A})\subseteq \bigcup_{i\in{S},j\in{\bar{S}}}\Upsilon_{i}^{j}(\mathcal{A})\subseteq \Upsilon^{S}(\mathcal{A})$.

(ii) $|x_{p}|\leq|x_{q}|$, so $|x_{q}|=\max_{i\in{N}}\{|x_{i}|\}$ and $|x_{q}|>0$. For any $i\in{S}$, it follows from (2) that

$$ \left \{ \textstyle\begin{array}{l} \sum_{i_{2},\ldots, i_{m}=1}^{n}a_{ii_{2}\cdots i_{m}}x_{i_{2}}\cdots x_{i_{m}}=\lambda x_{i}^{m-1}, \\ \sum_{i_{2},\ldots, i_{m}=1}^{n}a_{qi_{2}\cdots i_{m}}x_{i_{2}}\cdots x_{i_{m}}=\lambda x_{q}^{m-1}. \end{array}\displaystyle \right . $$

Using the same method as the proof in (i), we deduce that

$$\begin{aligned}& (\lambda-a_{i\cdots{i}})\sum_{\substack{\delta_{qi_{2}\cdots i_{m}=0}, \\ \delta_{ii_{2}\cdots i_{m}=0}}}a_{qi_{2}\cdots i_{m}}x_{i_{2}} \cdots x_{i_{m}} +a_{qi\cdots i}\sum_{\substack{\delta_{ii_{2}\cdots i_{m}=0}, \\ \delta _{qi_{2}\cdots i_{m}=0}}}a_{ii_{2}\cdots i_{m}}x_{i_{2}} \cdots x_{i_{m}} \\& \quad = (\lambda-a_{q\cdots{q}}) (\lambda-a_{i\cdots {i}})x_{q}^{m-1}-a_{iq\cdots{q}}a_{qi\cdots{i}}x_{q}^{m-1} \\& \quad = \bigl[(\lambda-a_{q\cdots{q}}) (\lambda-a_{i\cdots{i}})-a_{iq\cdots {q}}a_{qi\cdots{i}} \bigr]x_{q}^{m-1}. \end{aligned}$$

Taking the modulus in the above equation and using the triangle inequality we obtain

$$\begin{aligned}& \bigl\vert (\lambda-a_{q\cdots{q}}) (\lambda-a_{i\cdots{i}})-a_{iq\cdots {q}}a_{qi\cdots{i}} \bigr\vert \vert x_{q}\vert ^{m-1} \\& \quad \leq \vert \lambda-a_{i\cdots i}\vert r_{q}^{i}( \mathcal {A})\vert x_{q}\vert ^{m-1}+\vert a_{qi\cdots i}\vert r_{i}^{q}(\mathcal {A})\vert x_{q}\vert ^{m-1}. \end{aligned}$$

Note that $|x_{q}|>0$, thus

6 $$ \bigl\vert (\lambda-a_{q\cdots{q}}) (\lambda-a_{i\cdots{i}})-a_{iq\cdots {q}}a_{qi\cdots{i}} \bigr\vert \leq \vert \lambda-a_{i\cdots i}\vert r_{q}^{i}( \mathcal{A})+\vert a_{qi\cdots i}\vert r_{i}^{q}( \mathcal{A}). $$

This means that $\lambda\in\Upsilon_{q}^{i}(\mathcal{A})\subseteq \bigcup_{i\in{\bar{S}},j\in{S}}\Upsilon_{i}^{j}(\mathcal{A})\subseteq \Upsilon^{S}(\mathcal{A})$. This completes our proof of Theorem 3.1. □

#### Remark 3.1

Note that $|S|< n$, where $|S|$ is the cardinality of *S*. If $n=2$, then $|S|=1$ and $n(n-1)=2|S|(n-|S|)=2$, which implies that

$$ \Upsilon^{S}(\mathcal{A})= \bigl(\Upsilon_{1}^{2}( \mathcal{A})\cup \Upsilon_{2}^{1}(\mathcal{A}) \bigr)= \Delta(\mathcal{A}). $$

Besides, if $n\geq{3}$, $2|S|(n-|S|)< n(n-1)$, then $\Upsilon ^{S}(\mathcal{A})\subset\Delta(\mathcal{A})$ if $\Delta _{i_{1}}^{j_{1}}(\mathcal{A})\cap\Delta_{i_{2}}^{j_{2}}(\mathcal {A})=\varnothing$ for any $i_{1},i_{2},j_{1},j_{2}\in{N}$, $i_{1}\neq {i_{2}}$ or $j_{1}\neq{j_{2}}$. Furthermore, how to choose *S* to make $\Upsilon^{S}(\mathcal{A})$ as sharp as possible is very interesting and important. However, this work is difficult especially the dimension of the tensor $\mathcal{A}$ is large. At present, it is very difficult for us to research this problem, we will continue to study this problem in the future.

Next, we establish a comparison theorem for the new *S*-type eigenvalue inclusion set derived in this paper and those in Lemmas 1.1-1.4.

#### Theorem 3.2

*Let* $\mathcal{A}=(a_{i_{1}\cdots i_{m}})\in{\mathbb{C}}^{[m,n]}$ *with* $n\geq{2}$. *Then*

7 $$ \Upsilon^{S}(\mathcal{A})\subseteq\mathcal{K}^{S}( \mathcal {A})\subseteq\mathcal{K}(\mathcal{A})\subseteq\Gamma(\mathcal{A}),\qquad \Upsilon^{S}(\mathcal{A})\subseteq\Delta(\mathcal{A}). $$

#### Proof

According to Remark 3.1, it is obvious that $\Upsilon ^{S}(\mathcal{A})\subseteq\Delta(\mathcal{A})$. By Theorem 2.3 in [2], we know that $\mathcal{K}^{S}(\mathcal{A})\subseteq\mathcal {K}(\mathcal{A})\subseteq\Gamma(\mathcal{A})$. Hence, we only prove $\Upsilon^{S}(\mathcal{A})\subseteq\mathcal{K}^{S}(\mathcal{A})$. Let $z\in{\Upsilon^{S}(\mathcal{A})}$, then

$$ z\in\bigcup_{i\in{S},j\in{\bar{S}}}\Upsilon_{i}^{j}( \mathcal{A}) \quad \text{or}\quad z\in\bigcup_{i\in{\bar{S}},j\in{S}} \Upsilon _{i}^{j}(\mathcal{A}). $$

Without loss of generality, we assume that $z\in\bigcup_{i\in{S},j\in {\bar{S}}}\Upsilon_{i}^{j}(\mathcal{A})$ (we can prove it similarly if $z\in\bigcup_{i\in{\bar{S}},j\in{S}}\Upsilon_{i}^{j}(\mathcal{A})$). Then there exist $p\in{S}$ and $q\in{\bar{S}}$ such that $z\in\Upsilon _{p}^{q}(\mathcal{A})$, that is,

$$ \bigl\vert (z-a_{p\cdots p}) (z-a_{q\cdots q})-a_{pq\cdots q}a_{qp\cdots p} \bigr\vert \leq \vert z-a_{q\cdots q}\vert r_{p}^{q}( \mathcal{A})+\vert a_{pq\cdots q}\vert r_{q}^{p}( \mathcal{A}). $$

Inasmuch as

$$ \bigl\vert (z-a_{p\cdots p}) (z-a_{q\cdots q})\bigr\vert -\vert a_{pq\cdots q}a_{qp\cdots p}\vert \leq \bigl\vert (z-a_{p\cdots p}) (z-a_{q\cdots q})-a_{pq\cdots q}a_{qp\cdots p}\bigr\vert , $$

*z* satisfies

$$ \bigl\vert (z-a_{p\cdots p}) (z-a_{q\cdots q})\bigr\vert -\vert a_{pq\cdots q}a_{qp\cdots p}\vert \leq \vert z-a_{q\cdots q}\vert r_{p}^{q}(\mathcal{A})+\vert a_{pq\cdots q}\vert r_{q}^{p}(\mathcal{A}), $$

which yields

$$ \vert z-a_{q\cdots q}\vert \bigl(\vert z-a_{p\cdots p}\vert -r_{p}^{q}(\mathcal{A})\bigr)\leq \vert a_{pq\cdots q} \vert \bigl(r_{q}^{p}(\mathcal{A})+\vert a_{qp\cdots p}\vert \bigr)=\vert a_{pq\cdots q}\vert r_{q}( \mathcal{A}). $$

This means that

$$ z\in\mathcal{K}_{p,q}(A)\subseteq\bigcup_{i\in{S},j\in{\bar {S}}} \mathcal{K}_{i,j}(\mathcal{A})\subseteq\mathcal{K}^{S}(\mathcal {A}), $$

which implies that

$$ \Upsilon^{S}(\mathcal{A})\subseteq\mathcal{K}^{S}(\mathcal {A}). $$

This proof is completed. □

### A new upper bound for the spectral radius of nonnegative tensors

Based on the results of Section 3.1, we discuss the spectral radius of nonnegative tensors, and we give their upper bounds, which are better than those of Theorem 3.4 in [2].

#### Theorem 3.3

*Let* $\mathcal{A}\in{\mathbb{R}}^{[m,n]}$ *be an irreducible nonnegative tensor with* $n\geq{2}$. *And let* *S* *be a nonempty proper subset of* *N*. *Then*

$$ \rho(\mathcal{A})\leq\eta_{s}(\mathcal{A})=\max\bigl\{ \eta^{S}(\mathcal {A}),\eta^{\bar{S}}(\mathcal{A})\bigr\} , $$

*where*

8 $$ \eta^{S}(\mathcal{A}) =\frac{1}{2}\max _{i\in{S}}\min_{j\in{\bar{S}}}\bigl\{ a_{i\cdots i}+a_{j\cdots j}+r_{i}^{j}( \mathcal{A})+\Phi_{i,j}^{\frac {1}{2}}(\mathcal{A})\bigr\} , $$

*with*

$$ \Phi_{i,j}(\mathcal{A})=\bigl(a_{i\cdots i}-a_{j\cdots j}+r_{i}^{j}( \mathcal {A})\bigr)^{2}+4a_{ij\cdots{j}}r_{j}(\mathcal{A}). $$

#### Proof

Since $\mathcal{A}$ is an irreducible nonnegative tensor, by Lemma 2.1, there exists $x=(x_{1},\ldots,x_{n})^{T}>{0}$ such that

9 $$ \mathcal{A}x^{m-1}=\rho(\mathcal{A})x^{[m-1]}. $$

Let $x_{p}=\max_{i\in{S}}\{x_{i}\}$ and $x_{q}=\max_{i\in{\bar{S}}}\{ x_{i}\}$. Below we distinguish two cases to prove.

(i) $x_{p}\geq x_{q}>0$, so $x_{p}=\max_{i\in{N}}\{x_{i}\}$. For any $j\in{\bar{S}}$, it follows from (9) that

$$ \left \{ \textstyle\begin{array}{l} \sum_{i_{2},\ldots, i_{m}=1}^{n}a_{pi_{2}\cdots i_{m}}x_{i_{2}}\cdots x_{i_{m}}=\rho(\mathcal{A}) x_{p}^{m-1}, \\ \sum_{i_{2},\ldots, i_{m}=1}^{n}a_{ji_{2}\cdots i_{m}}x_{i_{2}}\cdots x_{i_{m}}=\rho(\mathcal{A}) x_{j}^{m-1}. \end{array}\displaystyle \right . $$

Hence, we have

10 $$ \left \{ \textstyle\begin{array}{l} \sum_{\substack{\delta_{pi_{2}\cdots i_{m}=0}, \\ \delta _{ji_{2}\cdots i_{m}=0}}}a_{pi_{2}\cdots i_{m}}x_{i_{2}}\cdots x_{i_{m}}=(\rho(\mathcal{A})-a_{p\cdots{p}})x_{p}^{m-1}-a_{pj\cdots {j}}x_{j}^{m-1}, \\ \sum_{\substack{\delta_{ji_{2}\cdots i_{m}=0}, \\ \delta _{pi_{2}\cdots i_{m}=0}}}a_{ji_{2}\cdots i_{m}}x_{i_{2}}\cdots x_{i_{m}}=(\rho(\mathcal{A})-a_{j\cdots{j}})x_{j}^{m-1}-a_{jp\cdots {p}}x_{p}^{m-1}. \end{array}\displaystyle \right . $$

Premultiplying by $(\rho(\mathcal{A})-a_{j\cdots{j}})$ in the first equation of (10) results in

11 $$\begin{aligned}& \bigl(\rho(\mathcal{A})-a_{j\cdots{j}}\bigr)\sum _{\substack{\delta _{pi_{2}\cdots i_{m}=0}, \\ \delta_{ji_{2}\cdots i_{m}=0}}}a_{pi_{2}\cdots i_{m}}x_{i_{2}}\cdots x_{i_{m}} \\& \quad = \bigl(\rho(\mathcal{A})-a_{j\cdots{j}}\bigr) \bigl(\rho( \mathcal{A})-a_{p\cdots {p}}\bigr)x_{p}^{m-1}-a_{pj\cdots{j}} \bigl(\rho(\mathcal{A})-a_{j\cdots{j}}\bigr)x_{j}^{m-1}. \end{aligned}$$

It follows from (11) and the second equation of (10) that

$$\begin{aligned}& \bigl(\rho(\mathcal{A})-a_{j\cdots{j}}\bigr)\sum_{\substack{\delta _{pi_{2}\cdots i_{m}=0}, \\ \delta_{ji_{2}\cdots i_{m}=0}}}a_{pi_{2}\cdots i_{m}}x_{i_{2}} \cdots x_{i_{m}} +a_{pj\cdots j}\sum_{\substack{\delta_{ji_{2}\cdots i_{m}=0}, \\ \delta _{pi_{2}\cdots i_{m}=0}}}a_{ji_{2}\cdots i_{m}}x_{i_{2}} \cdots x_{i_{m}} \\& \quad = \bigl(\rho(\mathcal{A})-a_{j\cdots{j}}\bigr) \bigl(\rho( \mathcal{A})-a_{p\cdots {p}}\bigr)x_{p}^{m-1}-a_{pj\cdots{j}}a_{jp\cdots{p}}x_{p}^{m-1} \\& \quad = \bigl[\bigl(\rho(\mathcal{A})-a_{j\cdots{j}}\bigr) \bigl(\rho( \mathcal{A})-a_{p\cdots {p}}\bigr)-a_{pj\cdots{j}}a_{jp\cdots{p}} \bigr]x_{p}^{m-1}. \end{aligned}$$

Note that $x_{p}\geq x_{j}$ for any $j\in{\bar{S}}$ and by Lemma 2.2, we deduce that

$$ \bigl[\bigl(\rho(\mathcal{A})-a_{j\cdots{j}}\bigr) \bigl(\rho( \mathcal{A})-a_{p\cdots {p}}\bigr)-a_{pj\cdots{j}}a_{jp\cdots{p}}\bigr] \leq \bigl(\rho(\mathcal{A})-a_{j\cdots{j}}\bigr)r_{p}^{j}( \mathcal {A})+a_{pj\cdots j}r_{j}^{p}(\mathcal{A}), $$

*i.e.*,

12 $$ \rho(\mathcal{A})^{2}-\bigl(a_{p\cdots p}+a_{j\cdots j}+r_{p}^{j}( \mathcal {A})\bigr)\rho(\mathcal{A}) +a_{j\cdots j}\bigl(a_{p\cdots p}+r_{p}^{j}( \mathcal{A})\bigr)-a_{pj\cdots j}r_{j}(\mathcal{A})\leq{0}. $$

Solving the quadratic inequality (12) yields

13 $$ \rho(\mathcal{A})\leq\frac{1}{2}\bigl\{ a_{p\cdots p}+a_{j\cdots j}+r_{p}^{j}( \mathcal{A})+\Phi_{p,j}^{\frac{1}{2}}(\mathcal{A})\bigr\} . $$

It is not difficult to verify that (13) can be true for any $j\in{\bar {S}}$. Thus

$$ \rho(\mathcal{A})\leq\frac{1}{2}\min_{j\in{\bar{S}}}\bigl\{ a_{p\cdots p}+a_{j\cdots j}+r_{p}^{j}(\mathcal{A})+ \Phi_{p,j}^{\frac {1}{2}}(\mathcal{A})\bigr\} , $$

which implies that

14 $$ \rho(\mathcal{A})\leq\frac{1}{2}\max_{i\in{S}} \min_{j\in{\bar{S}}}\bigl\{ a_{i\cdots i}+a_{j\cdots j}+r_{i}^{j}( \mathcal{A})+\Phi_{i,j}^{\frac {1}{2}}(\mathcal{A})\bigr\} . $$

(ii) $x_{q}\geq x_{p}>0$, so $x_{q}=\max_{i\in{N}}\{x_{i}\}$. For any $i\in{S}$, it follows from (9) that

$$ \left \{ \textstyle\begin{array}{l} \sum_{i_{2},\ldots, i_{m}=1}^{n}a_{ii_{2}\cdots i_{m}}x_{i_{2}}\cdots x_{i_{m}}=\rho(\mathcal{A}) x_{i}^{m-1}, \\ \sum_{i_{2},\ldots, i_{m}=1}^{n}a_{qi_{2}\cdots i_{m}}x_{i_{2}}\cdots x_{i_{m}}=\rho(\mathcal{A}) x_{q}^{m-1}. \end{array}\displaystyle \right . $$

So we obtain

15 $$ \left \{ \textstyle\begin{array}{l} \sum_{\substack{\delta_{ii_{2}\cdots i_{m}=0}, \\ \delta _{qi_{2}\cdots i_{m}=0}}}a_{ii_{2}\cdots i_{m}}x_{i_{2}}\cdots x_{i_{m}}=(\rho(\mathcal{A})-a_{i\cdots{i}})x_{i}^{m-1}-a_{iq\cdots {q}}x_{q}^{m-1}, \\ \sum_{\substack{\delta_{qi_{2}\cdots i_{m}=0}, \\ \delta _{ii_{2}\cdots i_{m}=0}}}a_{qi_{2}\cdots i_{m}}x_{i_{2}}\cdots x_{i_{m}}=(\rho(\mathcal{A})-a_{q\cdots{q}})x_{q}^{m-1}-a_{qi\cdots {i}}x_{i}^{m-1}. \end{array}\displaystyle \right . $$

In a similar manner to the proof of (i)

$$ \bigl[\bigl(\rho(\mathcal{A})-a_{q\cdots{q}}\bigr) \bigl(\rho( \mathcal{A})-a_{i\cdots {i}}\bigr)-a_{iq\cdots{q}}a_{qi\cdots{i}}\bigr] \leq \bigl(\rho(\mathcal{A})-a_{i\cdots{i}}\bigr)r_{q}^{i}( \mathcal {A})+a_{qi\cdots i}r_{i}^{q}(\mathcal{A}), $$

*i.e.*,

16 $$ \rho(\mathcal{A})^{2}-\bigl(a_{i\cdots i}+a_{q\cdots q}+r_{q}^{i}( \mathcal {A})\bigr)\rho(\mathcal{A}) +a_{i\cdots i}\bigl(a_{q\cdots q}+r_{q}^{i}( \mathcal{A})\bigr)-a_{qi\cdots i}r_{i}(\mathcal{A})\leq{0}, $$

which yields

17 $$ \rho(\mathcal{A})\leq\frac{1}{2}\bigl\{ a_{q\cdots q}+a_{i\cdots i}+r_{q}^{i}( \mathcal{A})+\Phi_{q,i}^{\frac{1}{2}}(\mathcal{A})\bigr\} . $$

It is easy to see that (17) can be true for any $j\in{S}$. Thus

$$ \rho(\mathcal{A})\leq\frac{1}{2}\min_{j\in{S}}\bigl\{ a_{q\cdots q}+a_{j\cdots j}+r_{q}^{j}(\mathcal{A})+ \Phi_{q,j}^{\frac {1}{2}}(\mathcal{A})\bigr\} , $$

which implies that

18 $$ \rho(\mathcal{A})\leq\frac{1}{2}\max_{i\in{\bar{S}}} \min_{j\in{S}}\bigl\{ a_{i\cdots i}+a_{j\cdots j}+r_{i}^{j}( \mathcal{A})+\Phi_{i,j}^{\frac {1}{2}}(\mathcal{A})\bigr\} . $$

This completes our proof in this theorem. □

Next, we extend the results of Theorem 3.3 to general nonnegative tensors; without the condition of irreducibility, compare with Theorem 3.3.

#### Theorem 3.4

*Let* $\mathcal{A}\in{\mathbb{R}}^{[m,n]}$ *be a nonnegative tensor with* $n\geq{2}$. *And let* *S* *be a nonempty proper subset of* *N*. *Then*

19 $$ \rho(\mathcal{A})\leq\eta_{s}=\max\bigl\{ \eta^{S}(\mathcal{A}),\eta^{\bar {S}}(\mathcal{A})\bigr\} , $$

*where*

$$ \eta^{S}(\mathcal{A})=\frac{1}{2}\max_{i\in{S}} \min_{j\in{\bar{S}}}\bigl\{ a_{i\cdots i}+a_{j\cdots j}+r_{i}^{j}( \mathcal{A})+\Phi_{i,j}^{\frac {1}{2}}(\mathcal{A})\bigr\} , $$

*with*

$$ \Phi_{i,j}(\mathcal{A})=\bigl(a_{i\cdots i}-a_{j\cdots j}+r_{i}^{j}( \mathcal {A})\bigr)^{2}+4a_{ij\cdots{j}}r_{j}(\mathcal{A}). $$

#### Proof

Let $\mathcal{A}_{k}=\mathcal{A}+\frac{1}{k}\varepsilon $, where $k=1,2,\ldots$ , and *ε* denotes the tensor with every entry being 1. Then $\mathcal{A}_{k}$ is a sequence of positive tensors satisfying

$$ 0\leq\mathcal{A}< \cdots< \mathcal{A}_{k+1}< \mathcal{A}_{k}< \cdots < \mathcal{A}_{1}. $$

By Lemma 2.3, $\{\rho(\mathcal{A}_{k})\}$ is a monotone decreasing sequence with lower bound $\rho(\mathcal{A})$. So $\rho(\mathcal {A}_{k})$ has a limit. Let

20 $$ \lim_{k\rightarrow+\infty}\rho(\mathcal{A}_{k})= \lambda\geq\rho (\mathcal{A}). $$

By Lemma 2.1, we see that $\rho(\mathcal{A}_{k})$ is the eigenvalue of $\mathcal{A}_{k}$ with a positive eigenvector $y_{k}$, *i.e.*, $\mathcal{A}_{k}y_{k}^{m-1}=\rho(\mathcal {A}_{k})y_{k}^{[m-1]}$. In a manner similar to Theorem 2.3 in [13], we have

$$ \lim_{k\rightarrow+\infty}\rho(\mathcal{A}_{k})=\rho(\mathcal {A}). $$

And we denote $\Psi_{i,j}(\mathcal{A})=\frac{1}{2}\{a_{i\cdots i}+a_{j\cdots j}+r_{i}^{j} (\mathcal{A})+\Phi_{i,j}^{\frac {1}{2}}(\mathcal{A})\}$ ($i\in{S}$, $j\in{\bar{S}}$ or $i\in{\bar{S}}$, $j\in {{S}}$). Then

$$ \Psi_{i,j}(\mathcal{A}_{k})=\frac{1}{2}\biggl\{ a_{i\cdots i}+a_{j\cdots j}+\frac{2}{k}+r_{i}^{j}( \mathcal{A})+\frac{n^{m-1}-2}{k}+\Phi _{i,j}^{\frac{1}{2}}( \mathcal{A}_{k})\biggr\} , $$

where

$$ \Phi_{i,j}(\mathcal{A}_{k})= \biggl(a_{i\cdots i}-a_{j\cdots j}+r_{i}^{j}( \mathcal{A}) +\frac{n^{m-1}-2}{k} \biggr)^{2}+4 \biggl(a_{ij\cdots{j}}+ \frac{1}{k} \biggr) \biggl(r_{j}(\mathcal{A})+\frac{n^{m-1}-1}{k} \biggr). $$

As *m* and *n* are finite numbers, then by the properties of the sequence, it is easy to see that

$$ \lim_{k\rightarrow+\infty}\Psi_{i,j}(\mathcal{A}_{k})= \Psi _{i,j}(\mathcal{A}). $$

Furthermore, since $\mathcal{A}_{k}$ is an irreducible nonnegative tensor, it follows from Theorem 3.3 that

$$ \rho(\mathcal{A}_{k})\leq \max\bigl\{ \eta^{S}( \mathcal{A}_{k}),\eta^{\bar{S}}(\mathcal{A}_{k})\bigr\} . $$

Letting $k\rightarrow+\infty$ results in

$$ \rho(\mathcal{A})\leq\max\bigl\{ \eta^{S}(\mathcal{A}), \eta^{\bar{S}}(\mathcal {A})\bigr\} , $$

from which one may get the desired bound (19). □

#### Remark 3.2

Now, we compare the upper bound in Theorem 3.4 with that in Theorem 3.4 in [2]. It is not difficult to see that

$$\begin{aligned} \eta^{S}(\mathcal{A}) =&\frac{1}{2}\max_{i\in{S}} \min_{j\in{\bar{S}}}\bigl\{ a_{i\cdots i}+a_{j\cdots j}+r_{i}^{j}( \mathcal{A})+\Phi_{i,j}^{\frac {1}{2}}(\mathcal{A})\bigr\} \\ \leq&\frac{1}{2}\max_{i\in{S},j\in{\bar{S}}}\bigl\{ a_{i\cdots i}+a_{j\cdots j}+r_{i}^{j}( \mathcal{A})+\Phi_{i,j}^{\frac {1}{2}}(\mathcal{A})\bigr\} \end{aligned}$$

and

$$\begin{aligned} \eta^{\bar{S}}(\mathcal{A}) =&\frac{1}{2}\max_{i\in{\bar{S}}} \min_{j\in{S}}\bigl\{ a_{i\cdots i}+a_{j\cdots j}+r_{i}^{j}( \mathcal{A})+\Phi_{i,j}^{\frac {1}{2}}(\mathcal{A})\bigr\} \\ \leq&\frac{1}{2}\max_{i\in{\bar{S}},j\in{S}}\bigl\{ a_{i\cdots i}+a_{j\cdots j}+r_{i}^{j}( \mathcal{A})+\Phi_{i,j}^{\frac {1}{2}}(\mathcal{A})\bigr\} . \end{aligned}$$

This shows that the upper bound in Theorem 3.4 improves the corresponding one in Theorem 3.4 of [2].

We have showed that our bound is sharper than the existing one in [2]. Now we take an example to show the efficiency of the new upper bound established in this paper.

#### Example 3.1

Let $\mathcal{A}=(a_{ijk})\in{\mathbb{R}}^{[3,3]}$ be nonnegative with entries defined as follows: $a_{111}=a_{122}=a_{222}=a_{233}=a_{312}=a_{322}=a_{333}=1$, $a_{123}=a_{133}=a_{211}=2$, $a_{213}=3$, $a_{311}=20$, and the other $a_{ijk}=0$. It is easy to compute

$$\begin{aligned}& r_{1}(\mathcal{A})=5,\qquad r_{1}^{2}( \mathcal{A})=4,\qquad r_{1}^{3}(\mathcal {A})=3; \\& r_{2}(\mathcal{A})=6,\qquad r_{2}^{1}( \mathcal{A})=4,\qquad r_{2}^{3}(\mathcal {A})=5; \\& r_{3}(\mathcal{A})=22,\qquad r_{3}^{1}( \mathcal{A})=2,\qquad r_{3}^{2}(\mathcal {A})=21. \end{aligned}$$

We choose $S=\{1,2\}$. Evidently, $\bar{S}=\{3\}$. By Theorem 3.4 of [2], we have

$$\rho(\mathcal{A})\leq22.2819. $$

By Theorem 3.4, we obtain

$$\rho(\mathcal{A})\leq12.0499, $$

which means that the upper bound in Theorem 3.4 is much better than that in Theorem 3.4 of [2].

### New upper and lower bounds for the minimum *H*-eigenvalue of nonsingular *M*-tensors

In this section, by making use of the results in Section 3.1, we investigate the bounds for the minimum *H*-eigenvalue of strong *M*-tensors and derive sharper bounds for that. This bounds are proved to be tighter than those in Theorem 2.2 of [4].

#### Theorem 3.5

*Let* $\mathcal{A}\in{\mathbb{R}}^{[m,n]}$ *be an irreducible nonsingular* *M*-*tensor with* $n\geq{2}$. *And let* *S* *be a nonempty proper subset of* *N*. *Then*

$$ \min\bigl\{ \phi^{S}(\mathcal{A}),\phi^{\bar{S}}(\mathcal{A})\bigr\} \leq\tau (\mathcal{A})\leq\max\bigl\{ \chi^{S}(\mathcal{A}), \chi^{\bar{S}}(\mathcal {A})\bigr\} , $$

*where*

21 $$ \chi^{S}(\mathcal{A})=\frac{1}{2}\max _{i\in{S}}\min_{j\in{\bar{S}}}\bigl\{ a_{i\cdots i}+a_{j\cdots j}-r_{i}^{j}( \mathcal{A})-\Theta_{i,j}^{\frac {1}{2}}(\mathcal{A})\bigr\} $$

*and*

22 $$ \phi^{S}(\mathcal{A})=\frac{1}{2}\min _{i\in{S}}\max_{j\in{\bar{S}}}\bigl\{ a_{i\cdots i}+a_{j\cdots j}-r_{i}^{j}( \mathcal{A})-\Theta_{i,j}^{\frac {1}{2}}(\mathcal{A})\bigr\} , $$

*with*

$$ \Theta_{i,j}(\mathcal{A})=\bigl(a_{i\cdots i}-a_{j\cdots j}-r_{i}^{j}( \mathcal{A})\bigr)^{2}-4a_{ij\cdots{j}}r_{j}(\mathcal {A}). $$

#### Proof

Since $\mathcal{A}$ is an irreducible nonsingular *M*-tensor, by Lemma 2.4, there exists $x=(x_{1},\ldots,x_{n})^{T}>{0}$ such that

23 $$ \mathcal{A}x^{m-1}=\tau(\mathcal{A})x^{[m-1]}. $$

Let $x_{p}=\max_{i\in{S}}\{x_{i}\}$ and $x_{q}=\max_{i\in{\bar{S}}}\{ x_{i}\}$. We distinguish two cases to prove.

(i) $x_{p}\geq x_{q}>0$, so $x_{p}=\max_{i\in{N}}\{x_{i}\}$. For any $j\in{\bar{S}}$, it follows from (23) that

$$ \left \{ \textstyle\begin{array}{l} \sum_{i_{2},\ldots, i_{m}=1}^{n}a_{pi_{2}\cdots i_{m}}x_{i_{2}}\cdots x_{i_{m}}=\tau(\mathcal{A}) x_{p}^{m-1}, \\ \sum_{i_{2},\ldots, i_{m}=1}^{n}a_{ji_{2}\cdots i_{m}}x_{i_{2}}\cdots x_{i_{m}}=\tau(\mathcal{A}) x_{j}^{m-1}. \end{array}\displaystyle \right . $$

Hence, we have

24 $$ \left \{ \textstyle\begin{array}{l} -\sum_{\substack{\delta_{pi_{2}\cdots i_{m}=0}, \\ \delta _{ji_{2}\cdots i_{m}=0}}}a_{pi_{2}\cdots i_{m}}x_{i_{2}}\cdots x_{i_{m}}=(a_{p\cdots{p}}-\tau(\mathcal{A}))x_{p}^{m-1}+a_{pj\cdots {j}}x_{j}^{m-1}, \\ -\sum_{\substack{\delta_{ji_{2}\cdots i_{m}=0}, \\ \delta _{pi_{2}\cdots i_{m}=0}}}a_{ji_{2}\cdots i_{m}}x_{i_{2}}\cdots x_{i_{m}}=(a_{j\cdots{j}}-\tau(\mathcal{A}))x_{j}^{m-1}+a_{jp\cdots {p}}x_{p}^{m-1}. \end{array}\displaystyle \right . $$

Premultiplying by $(a_{j\cdots{j}}-\tau(\mathcal{A}))$ in the first equation of (24) results in

25 $$\begin{aligned}& -\bigl(a_{j\cdots{j}}-\tau(\mathcal{A})\bigr)\sum _{\substack{\delta _{pi_{2}\cdots i_{m}=0}, \\ \delta_{ji_{2}\cdots i_{m}=0}}}a_{pi_{2}\cdots i_{m}}x_{i_{2}}\cdots x_{i_{m}} \\& \quad = \bigl(a_{j\cdots{j}}-\tau(\mathcal{A})\bigr) \bigl(a_{p\cdots{p}}- \tau(\mathcal {A})\bigr)x_{p}^{m-1}+a_{pj\cdots{j}} \bigl(a_{j\cdots{j}}-\tau(\mathcal{A})\bigr)x_{j}^{m-1}. \end{aligned}$$

It follows from (25) and the second equation of (24) that

$$\begin{aligned}& -\bigl(a_{j\cdots{j}}-\tau(\mathcal{A})\bigr)\sum _{\substack{\delta _{pi_{2}\cdots i_{m}=0}, \\ \delta_{ji_{2}\cdots i_{m}=0}}}a_{pi_{2}\cdots i_{m}}x_{i_{2}}\cdots x_{i_{m}} +a_{pj\cdots j}\sum_{\substack{\delta_{ji_{2}\cdots i_{m}=0}, \\ \delta _{pi_{2}\cdots i_{m}=0}}}a_{ji_{2}\cdots i_{m}}x_{i_{2}} \cdots x_{i_{m}} \\& \quad = \bigl(a_{j\cdots{j}}-\tau(\mathcal{A})\bigr) \bigl(a_{p\cdots{p}}- \tau(\mathcal {A})\bigr)x_{p}^{m-1}-a_{pj\cdots{j}}a_{jp\cdots{p}}x_{p}^{m-1} \\& \quad = \bigl[\bigl(a_{j\cdots{j}}-\tau(\mathcal{A})\bigr) \bigl(a_{p\cdots{p}}-\tau(\mathcal {A})\bigr)-a_{pj\cdots{j}}a_{jp\cdots{p}} \bigr]x_{p}^{m-1}. \end{aligned}$$

Combining $x_{p}\geq x_{j}$ for any $j\in{\bar{S}}$ with Lemma 2.5 results in

$$ \bigl[\bigl(a_{j\cdots{j}}-\tau(\mathcal{A})\bigr) \bigl(a_{p\cdots{p}}- \tau(\mathcal {A})\bigr)-a_{pj\cdots{j}}a_{jp\cdots{p}}\bigr] \leq \bigl(a_{j\cdots{j}}-\tau(\mathcal{A})\bigr)r_{p}^{j}( \mathcal {A})-a_{pj\cdots j}r_{j}^{p}(\mathcal{A}), $$

*i.e.*,

26 $$ \tau(\mathcal{A})^{2}-\bigl(a_{p\cdots p}+a_{j\cdots j}-r_{p}^{j}( \mathcal {A})\bigr)\tau(\mathcal{A}) +a_{j\cdots j}\bigl(a_{p\cdots p}-r_{p}^{j}( \mathcal{A})\bigr)+a_{pj\cdots j}r_{j}(\mathcal{A})\leq{0}. $$

Solving the quadratic inequality (26) yields

27 $$ \tau(\mathcal{A})\geq\frac{1}{2}\bigl\{ a_{p\cdots p}+a_{j\cdots j}-r_{p}^{j}( \mathcal{A})-\Theta_{p,j}^{\frac{1}{2}}(\mathcal{A})\bigr\} . $$

It is not difficult to verify that (27) can be true for any $j\in{\bar {S}}$. Thus

$$ \tau(\mathcal{A})\geq\frac{1}{2}\max_{j\in{\bar{S}}}\bigl\{ a_{p\cdots p}+a_{j\cdots j}-r_{p}^{j}(\mathcal{A})- \Theta_{p,j}^{\frac {1}{2}}(\mathcal{A})\bigr\} , $$

and therefore

28 $$ \tau(\mathcal{A})\geq\frac{1}{2}\min_{i\in{S}} \max_{j\in{\bar{S}}}\bigl\{ a_{i\cdots i}+a_{j\cdots j}-r_{i}^{j}( \mathcal{A})-\Theta_{i,j}^{\frac {1}{2}}(\mathcal{A})\bigr\} . $$

(ii) $x_{q}\geq x_{p}>0$, so $x_{q}=\max_{i\in{N}}\{x_{i}\}$. For any $i\in{S}$, it follows from (23) that

$$ \left \{ \textstyle\begin{array}{l} \sum_{i_{2},\ldots, i_{m}=1}^{n}a_{ii_{2}\cdots i_{m}}x_{i_{2}}\cdots x_{i_{m}}=\tau(\mathcal{A}) x_{i}^{m-1}, \\ \sum_{i_{2},\ldots, i_{m}=1}^{n}a_{qi_{2}\cdots i_{m}}x_{i_{2}}\cdots x_{i_{m}}=\tau(\mathcal{A}) x_{q}^{m-1}. \end{array}\displaystyle \right . $$

So we obtain

29 $$ \left \{ \textstyle\begin{array}{l} -\sum_{\substack{\delta_{ii_{2}\cdots i_{m}=0}, \\ \delta _{qi_{2}\cdots i_{m}=0}}}a_{ii_{2}\cdots i_{m}}x_{i_{2}}\cdots x_{i_{m}}=(a_{i\cdots{i}}-\tau(\mathcal{A}))x_{i}^{m-1}+a_{iq\cdots {q}}x_{q}^{m-1}, \\ -\sum_{\substack{\delta_{qi_{2}\cdots i_{m}=0}, \\ \delta _{ii_{2}\cdots i_{m}=0}}}a_{qi_{2}\cdots i_{m}}x_{i_{2}}\cdots x_{i_{m}}=(a_{q\cdots{q}}-\tau(\mathcal{A}))x_{q}^{m-1}+a_{qi\cdots {i}}x_{i}^{m-1}. \end{array}\displaystyle \right . $$

Using the same technique as the proof of (i), we have

$$ \bigl[\bigl(a_{q\cdots{q}}-\tau(\mathcal{A})\bigr) \bigl(a_{i\cdots{i}}- \tau(\mathcal {A})\bigr)-a_{iq\cdots{q}}a_{qi\cdots{i}}\bigr] \leq \bigl(a_{i\cdots{i}}-\tau(\mathcal{A})\bigr)r_{q}^{i}( \mathcal {A})-a_{qi\cdots i}r_{i}^{q}(\mathcal{A}), $$

which is equivalent to

30 $$ \tau(\mathcal{A})^{2}-\bigl(a_{q\cdots q}+a_{i\cdots i}-r_{q}^{i}( \mathcal {A})\bigr)\tau(\mathcal{A}) +a_{i\cdots i}\bigl(a_{q\cdots q}-r_{q}^{i}( \mathcal{A})\bigr)+a_{qi\cdots i}r_{i}(\mathcal{A})\leq{0}, $$

which results in

31 $$ \tau(\mathcal{A})\geq\frac{1}{2}\bigl\{ a_{q\cdots q}+a_{i\cdots i}-r_{q}^{i}( \mathcal{A})-\Theta_{q,i}^{\frac{1}{2}}(\mathcal{A})\bigr\} . $$

It is not difficult to verify that (31) can be true for any $j\in{S}$. Thus

$$ \tau(\mathcal{A})\geq\frac{1}{2}\max_{j\in{S}}\bigl\{ a_{q\cdots q}+a_{j\cdots j}-r_{q}^{j}(\mathcal{A})- \Theta_{q,j}^{\frac {1}{2}}(\mathcal{A})\bigr\} , $$

which implies that

$$ \tau(\mathcal{A})\geq\frac{1}{2}\min_{i\in{\bar{S}}}\max _{j\in{S}}\bigl\{ a_{i\cdots i}+a_{j\cdots j}-r_{i}^{j}( \mathcal{A})-\Theta_{i,j}^{\frac {1}{2}}(\mathcal{A})\bigr\} . $$

Let $x_{k}=\min_{i\in{S}}\{x_{i}\}$ and $x_{l}=\min_{i\in{\bar{S}}}\{ x_{i}\}$. With a strategy quite similar to the one utilized in the above proof, we can prove that

$$ \tau(\mathcal{A})\leq\max\bigl\{ \chi^{S}(\mathcal{A}), \chi^{\bar{S}}(\mathcal {A})\bigr\} , $$

which implies this theorem. □

#### Remark 3.3

We next prove the bounds in Theorem 3.5 are sharper than those of Theorem 2.2 in [4]; it is easy to see that

$$\begin{aligned} \phi^{S}(\mathcal{A}) =&\frac{1}{2}\min_{i\in{S}} \max_{j\in{\bar{S}}}\bigl\{ a_{i\cdots i}+a_{j\cdots j}-r_{i}^{j}( \mathcal{A})-\Theta_{i,j}^{\frac {1}{2}}(\mathcal{A})\bigr\} \\ \geq&\frac{1}{2}\min_{i\in{S},j\in{\bar{S}}}\bigl\{ a_{i\cdots i}+a_{j\cdots j}-r_{i}^{j}( \mathcal{A})-\Theta_{i,j}^{\frac {1}{2}}(\mathcal{A})\bigr\} = \psi^{S}(\mathcal{A}) \end{aligned}$$

and

$$\begin{aligned} \phi^{\bar{S}}(\mathcal{A}) =&\frac{1}{2}\min_{i\in{\bar{S}}} \max_{j\in {{S}}}\bigl\{ a_{i\cdots i}+a_{j\cdots j}-r_{i}^{j}( \mathcal{A})-\Theta _{i,j}^{\frac{1}{2}}(\mathcal{A})\bigr\} \\ \geq&\frac{1}{2}\min_{i\in{\bar{S}},j\in{S}}\bigl\{ a_{i\cdots i}+a_{j\cdots j}-r_{i}^{j}( \mathcal{A})-\Theta_{i,j}^{\frac {1}{2}}(\mathcal{A})\bigr\} = \psi^{\bar{S}}(\mathcal{A}), \end{aligned}$$

which implies that

$$\begin{aligned} \min\bigl\{ \phi^{S}(\mathcal{A}),\phi^{\bar{S}}(\mathcal{A})\bigr\} \geq&\min\bigl\{ \psi^{S}(\mathcal{A}),\psi^{\bar{S}}( \mathcal{A})\bigr\} \\ \geq&\frac{1}{2}\min_{i,j\in{N},i\neq{j}}\bigl\{ a_{i\cdots i}+a_{j\cdots j}-r_{i}^{j}( \mathcal{A})-\Theta_{i,j}^{\frac{1}{2}}(\mathcal{A})\bigr\} . \end{aligned}$$

In the same manner as applied in the above proof, we can deduce the following results:

$$\begin{aligned} \max\bigl\{ \chi^{S}(\mathcal{A}),\chi^{\bar{S}}(\mathcal{A})\bigr\} \leq&\max\bigl\{ \theta^{S}(\mathcal{A}),\theta^{\bar{S}}( \mathcal{A})\bigr\} \\ \leq&\frac{1}{2}\max_{i,j\in{N},i\neq{j}}\bigl\{ a_{i\cdots i}+a_{j\cdots j}-r_{i}^{j}( \mathcal{A})-\Theta_{i,j}^{\frac{1}{2}}(\mathcal{A})\bigr\} , \end{aligned}$$

where $\theta^{S}(\mathcal{A})=\frac{1}{2}\max_{i\in{S},j\in {\bar{S}}}\{a_{i\cdots i}+a_{j\cdots j}-r_{i}^{j}(\mathcal{A})-\Theta _{i,j}^{\frac{1}{2}}(\mathcal{A})\}$. Therefore, the conclusions follow from the above discussions.

#### Example 3.2

Consider the following irreducible nonsingular *M*-tensor:

$$ \mathcal{A}=\bigl[A(1,:,:),A(2,:,:),A(3,:,:)\bigr]\in{\mathbb {R}}^{[3,3]}, $$

where

$$\begin{aligned}& A(1,:,:)=\left ( \textstyle\begin{array}{@{}c@{\quad}c@{\quad}c@{}} 7 & 0 & 0 \\ 0 & -0.5 & -2 \\ 0 & -1 & -2 \end{array}\displaystyle \right ), \\& A(2,:,:)=\left ( \textstyle\begin{array}{@{}c@{\quad}c@{\quad}c@{}} -1 & -5.8 & -2 \\ 0 & 12 & 0 \\ 0 & 0 & -0.5 \end{array}\displaystyle \right ), \\& A(3,:,:)=\left ( \textstyle\begin{array}{@{}c@{\quad}c@{\quad}c@{}} -1 & -2 & 0 \\ 0 & -1 & -3 \\ 0 & -3 & 50 \end{array}\displaystyle \right ). \end{aligned}$$

We compare the results derived in Theorem 3.5 with those in Theorem 2.1 of [4] and Theorem 4.5 of [16] in the correct forms. Let $S=\{ 1,2\}$, then $\bar{S}=\{3\}$. By Theorem 2.1 of [4], we have

$$1.5548\leq\tau(\mathcal{A})\leq11.6828. $$

By Theorem 4.5 of [16], we get

$$1.7350\leq\tau(\mathcal{A})\leq11.3923. $$

By Theorem 3.5, we obtain

$$3.0738\leq\tau(\mathcal{A})\leq6.8390. $$

This shows that the upper and lower bounds in Theorem 3.5 are sharper than those in Theorem 2.1 of [4] and Theorem 4.5 of [16].

In the sequel, we extend the results of Theorem 3.5 to a more general case, which needs a weaker condition compared with Theorem 3.5.

#### Theorem 3.6

*Let* $\mathcal{A}\in{\mathbb{R}}^{[m,n]}$ *be a nonsingular* *M*-*tensor with* $n\geq{2}$. *And let* *S* *be a nonempty proper subset of* *N*. *Then*

$$ \min\bigl\{ \phi^{S}(\mathcal{A}),\phi^{\bar{S}}(\mathcal{A})\bigr\} \leq\tau (\mathcal{A})\leq\max\bigl\{ \chi^{S}(\mathcal{A}), \chi^{\bar{S}}(\mathcal {A})\bigr\} , $$

*where*

32 $$ \chi^{S}(\mathcal{A})=\frac{1}{2}\max _{i\in{S}}\min_{j\in{\bar{S}}}\bigl\{ a_{i\cdots i}+a_{j\cdots j}-r_{i}^{j}( \mathcal{A})-\Theta_{i,j}^{\frac {1}{2}}(\mathcal{A})\bigr\} , $$

*and*

33 $$ \phi^{S}(\mathcal{A})=\frac{1}{2}\min _{i\in{S}}\max_{j\in{\bar{S}}}\bigl\{ a_{i\cdots i}+a_{j\cdots j}-r_{i}^{j}( \mathcal{A})-\Theta_{i,j}^{\frac {1}{2}}(\mathcal{A})\bigr\} , $$

*with*

$$ \Theta_{i,j}(\mathcal{A})=\bigl(a_{i\cdots i}-a_{j\cdots j}-r_{i}^{j}( \mathcal{A})\bigr)^{2}-4a_{ij\cdots{j}}r_{j}(\mathcal {A}). $$

#### Proof

Since $\mathcal{A}$ is a nonsingular *M*-tensor, $\mathcal{A}\in{\mathbb {Z}}$, by Lemma 2.7 and Lemma 2.6, there exists $x=(x_{1},\ldots ,x_{n})^{T}\geq{0}$ such that $\mathcal{A}x^{m-1}>0$, that is, for any $i_{1}\in{N}$,

$$ \sum_{i_{2},\ldots,i_{m}=1}^{n}a_{i_{1}\cdots i_{m}}x_{i_{2}} \cdots x_{i_{m}}>0. $$

Let

$$ g=\min_{i_{1}\in{N}}\sum_{i_{2},\ldots, i_{m}=1}^{n}a_{i_{1}\cdots i_{m}}x_{i_{2}} \cdots x_{i_{m}}, x_{\mathrm{max}}=\max_{i\in{N}}x_{i}. $$

So $x_{\mathrm{max}}>0$ by $x\geq{0}$. By replacing the zero entries of $\mathcal {A}$ with $-\frac{1}{k}$, where *k* is a positive integer, we see that the *Z*-tensor $\mathcal{A}_{k}$ is irreducible. Here, we use $a_{i_{1}\cdots i_{m}}(-\frac{1}{k})$ to denote the entries of $\mathcal {A}_{k}$. We choose $k>[\frac{(n^{m-1}-1)x_{\mathrm{max}}^{m-1}}{g}]+1$, then, for any $i_{1}\in{N}$, we have

$$\begin{aligned} \begin{aligned} &\sum_{i_{2},\ldots, i_{m}=1}^{n}a_{i_{1}\cdots i_{m}}\biggl(- \frac {1}{k}\biggr)x_{i_{2}}\cdots x_{i_{m}} \\ &\quad \geq \min_{i_{1}\in{N}}\sum_{i_{2},\ldots, i_{m}=1}^{n}a_{i_{1}\cdots i_{m}}x_{i_{2}} \cdots x_{i_{m}}-\frac {(n^{m-1}-1)x_{\mathrm{max}}^{m-1}}{k} \\ &\quad = g-\frac{(n^{m-1}-1)x_{\mathrm{max}}^{m-1}}{k}>0, \end{aligned} \end{aligned}$$

which implies that $\mathcal{A}_{k}x^{m-1}>0$ and, by Lemma 2.6 and Lemma 2.7, we infer that $\mathcal{A}_{k}$ is an irreducible nonsingular *M*-tensor if $k>[\frac{(n^{m-1}-1)x_{\mathrm{max}}^{m-1}}{g}]+1$. It follows from the above discussions that $\mathcal{A}_{k}$ ($k>[\frac {(n^{m-1}-1)x_{\mathrm{max}}^{m-1}}{g}]+1$) is a sequence of irreducible nonsingular *M*-tensors satisfying

$$ \mathcal{A}>\cdots>\mathcal{A}_{k+1}>\mathcal{A}_{k}. $$

By Lemma 2.8, $\{\tau(\mathcal{A}_{k})\}$ is a monotone increasing sequence with upper bound $\tau(\mathcal{A})$ so that $\tau(\mathcal {A}_{k})$ has a limit. Let

34 $$ \lim_{k\rightarrow+\infty}\tau(\mathcal{A}_{k})= \lambda\leq\tau (\mathcal{A}). $$

By Lemma 2.4, we see that $\tau(\mathcal{A}_{k})$ is the eigenvalue of $\mathcal{A}_{k}$ with a positive eigenvector $y_{k}$, *i.e.*, $\mathcal{A}_{k}y_{k}^{m-1}=\tau(\mathcal {A}_{k})y_{k}^{[m-1]}$. As homogeneous multivariable polynomials, we can restrict $y_{k}$ to a ball; that is, $\|y_{k}\|=1$. Then $\{y_{k}\} $ is a bounded sequence, so it has a convergent subsequence. Without loss of generality, we can suppose it is the sequence itself. Let $y_{k}\rightarrow{y}$ as $k\rightarrow+\infty$, we get $y\geq{0}$ and $\|y\|=1$. By $\mathcal{A}_{k}y_{k}^{m-1}=\tau(\mathcal {A}_{k})y_{k}^{[m-1]}$ and letting $k\rightarrow+\infty$, we have $\mathcal{A}y=\lambda y^{[m-1]}$. Thus *λ* is an eigenvalue of $\mathcal{A}$, thus $\lambda\geq\tau(\mathcal{A})$. Together with (34) this results in $\lambda=\tau(\mathcal{A})$, which means that

$$ \lim_{k\rightarrow+\infty}\tau(\mathcal{A}_{k})=\tau(\mathcal {A}). $$

Besides, for $i\in{S}$, $j\in{\bar{S}}$ (for $i\in{\bar{S}}$, $j\in{S}$, we can define $M_{i}^{j}$ and $M_{j}$ similarly), we define the following sets:

$$\begin{aligned}& M_{i}^{j}=\{a_{ii_{2}\cdots i_{m}}|a_{ii_{2}\cdots i_{m}}=0, \delta _{ii_{2}\cdots i_{m}}=0 \text{ and } \delta_{ji_{2}\cdots i_{m}}=0, i_{2}, \ldots,i_{m}\in{N}\}, \\& M_{j}=\{a_{ji_{2}\cdots i_{m}}|a_{ji_{2}\cdots i_{m}}=0 \text{ and } \delta_{ji_{2}\cdots i_{m}}=0, i_{2},\ldots,i_{m}\in{N}\}. \end{aligned}$$

Let the numbers of entries in $M_{i}^{j}$ and $M_{j}$ be $n_{i}^{j}$ and $n_{j}$, respectively, and we denote $\Lambda_{i,j}(\mathcal {A})=\frac{1}{2}\{a_{i\cdots i}+a_{j\cdots j}-r_{i}^{j}(\mathcal {A})-\Theta_{i,j}^{\frac{1}{2}}(\mathcal{A})\}$. Then

$$ \Lambda_{i,j}(\mathcal{A}_{k})=\frac{1}{2}\biggl\{ a_{i\cdots i}+a_{j\cdots j}-r_{i}^{j}(\mathcal{A})- \frac{n_{i}^{j}}{k}-\Theta_{i,j}^{\frac {1}{2}}(\mathcal{A}_{k}) \biggr\} , $$

where

$$ \Theta_{i,j}(\mathcal{A}_{k})= \biggl(a_{i\cdots i}-a_{j\cdots j}-r_{i}^{j}( \mathcal{A})-\frac{n_{i}^{j}}{k} \biggr)^{2}-4 \biggl(a_{ij\cdots {j}}- \frac{\varepsilon_{i,j}}{k} \biggr) \biggl(r_{j}(\mathcal{A})+\frac {n_{j}}{k} \biggr), $$

with

$$ \varepsilon_{i,j}=\left \{ \textstyle\begin{array}{l@{\quad}l} 1,& \text{if } a_{ij\cdots{j}}=0, \\ 0,& \text{if } a_{ij\cdots{j}}\neq{0}. \end{array}\displaystyle \right . $$

By the properties of the sequence, it is not difficult to verify that

$$ \lim_{k\rightarrow+\infty}\chi^{S}(\mathcal{A}_{k})= \chi^{S}(\mathcal {A}), \qquad \lim_{k\rightarrow+\infty} \phi^{S}(\mathcal{A}_{k})=\phi ^{S}(\mathcal{A}). $$

Furthermore, since $\mathcal{A}_{k}$ is an irreducible nonsingular *M*-tensor for $k>[\frac{(n^{m-1}-1)x_{\mathrm{max}}^{m-1}}{g}]+1$, by Theorem 3.5, we have

$$ \min\bigl\{ \phi^{S}(\mathcal{A}_{k}),\phi^{\bar{S}}( \mathcal{A}_{k})\bigr\} \leq \tau(\mathcal{A}_{k})\leq\max \bigl\{ \chi^{S}(\mathcal{A}_{k}),\chi^{\bar {S}}( \mathcal{A}_{k})\bigr\} . $$

Letting $k\rightarrow+\infty$ results in

$$ \min\bigl\{ \phi^{S}(\mathcal{A}),\phi^{\bar{S}}(\mathcal{A})\bigr\} \leq\tau (\mathcal{A})\leq\max\bigl\{ \chi^{S}(\mathcal{A}), \chi^{\bar{S}}(\mathcal {A})\bigr\} . $$

This completes our proof of Theorem 3.6. □

## Concluding remarks

In this paper, a new *S*-type eigenvalue inclusion set for tensors is presented, which is proved to be sharper than the ones in [2, 3]. As applications, we give new bounds for the spectral radius of nonnegative tensors and the minimum *H*-eigenvalue of strong *M*-tensors, these bounds improve some existing ones obtained by Li *et al.* [2] and He and Huang [4]. In addition, we extend these new bounds to more general cases.

However, the new *S*-type eigenvalue inclusion set and the derived bounds depend on the set *S*. How to choose *S* to make $\Upsilon ^{S}(\mathcal{A})$ and the bounds exhibited in this paper as tight as possible is very important and interesting, while if the dimension of the tensor $\mathcal{A}$ is large, this work is very difficult. Therefore, future work will include numerical or theoretical studies for finding the best choice for *S*.
